# Supplementary material for: The interactome of intact mitochondria by cross-linking mass spectrometry provides evidence for coexisting respiratory supercomplexes
Source: Mol Cell Proteomics. 2017 Dec 8;17(2):216–32. doi: 10.1074/mcp.RA117.000470 (PMC5795388; doi:10.1074/mcp.RA117.000470)
Supplement: Supplemental Data [file supp_RA117.000470_133922_0_supp_23978_fzffwf.zip › spectra_annotation/mito_DR_spectra_annotation/326-1-5-1-4-1.pdf]

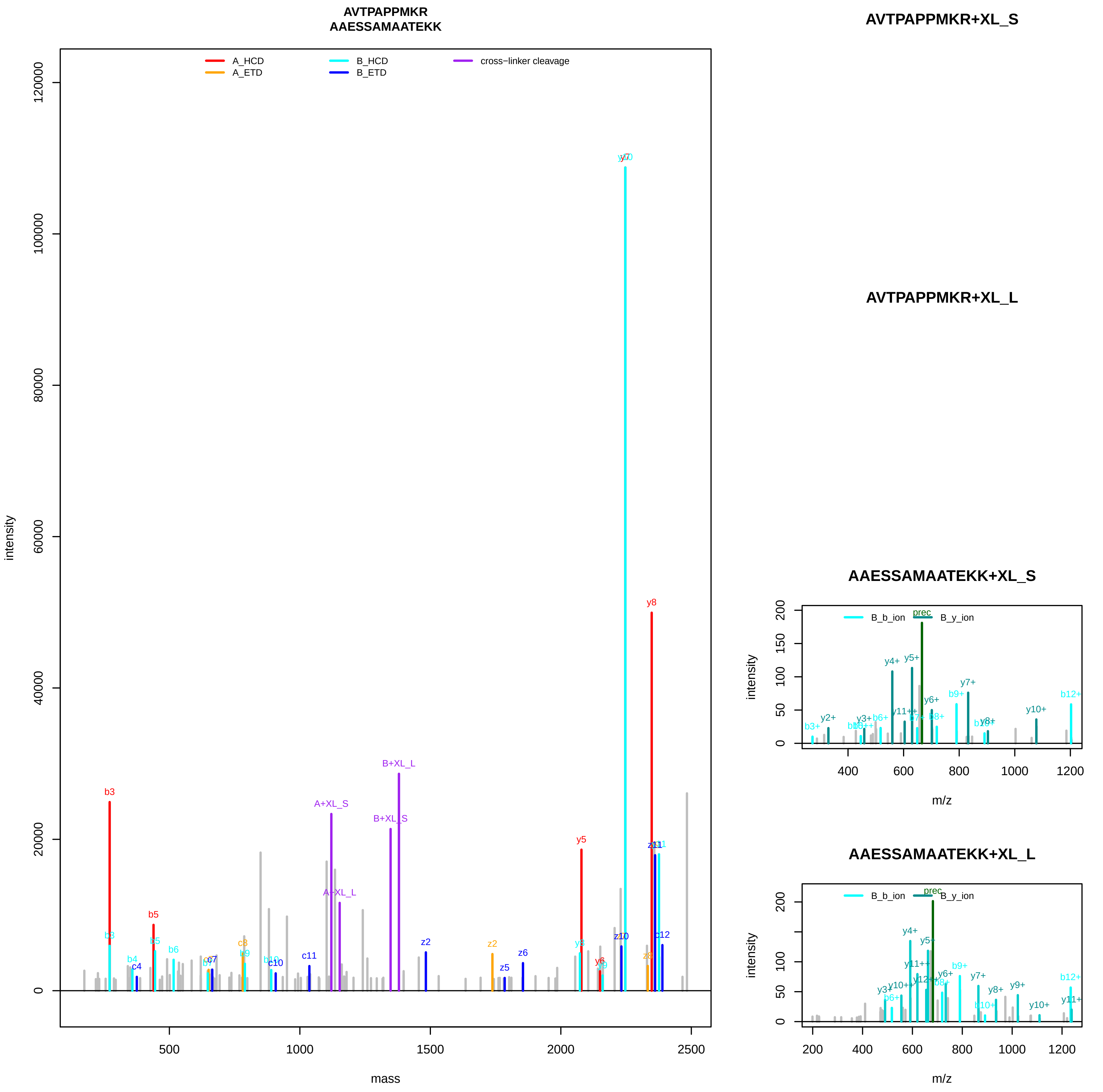
AVTPAPPMKR
AAESSAMAATEKK
A_HCD
A_ETD
B_HCD
B_ETD
cross−linker cleavage
intensity
mass
0
20000
40000
60000
80000
100000
120000
500
1000
1500
2000
2500
b3
b5
b4
c4
b6
c7
c8
b9
c10
c11
A+XL_S
A+XL_L
B+XL_S
B+XL_L
z2
z5
z6
y5
y6
z10
y7
y8
z11
c12
AVTPAPPMKR+XL_S
AVTPAPPMKR+XL_L
AAESSAMAATEKK+XL_S
B_b_ion
B_y_ion
prec
y2+
b3+
y3+
b6+
y4+
y5+
y11++
y6+
b9+
y7+
y8+
y10+
b12+
m/z
AAESSAMAATEKK+XL_L
y9+
b10+
y11+
0
50
100
150
200
200
400
600
800
1000
1200
